# Leave Against Medical Advice amongst Pediatric Patients Admitted in a Tertiary Teaching Hospital in Nepal: A Descriptive Cross-sectional Study

**DOI:** 10.31729/jnma.5757

**Published:** 2021-09-30

**Authors:** Sahisnuta Basnet, B.K. Ganesh, Aslam Ansari, Suraj Adhikari

**Affiliations:** 1 Department of Pediatrics, Manipal College of Medical Sciences, Phulbari, Pokhara, Nepal; 2 Manipal College of Medical Sciences, Phulbari, Pokhara, Nepal

**Keywords:** *children*, *discharge against medical advice*, *health insurance*

## Abstract

**Introduction:**

Leave against medical advice has a potentially deleterious effect on the health of a child. This is particularly alarming in case of pediatric patients as they are unable to understand the implications of it and rely on parents to make decisions regarding their health. This study was undertaken to find out the prevalence of leave against medical advice among pediatric patients admitted in a tertiary teaching hospital in Nepal.

**Methods:**

A descriptive cross-sectional study was conducted in the Department of Pediatrics, Manipal Teaching Hospital between August 2019 and July 2020. Ethical clearance was obtained from the Institutional Review Committee (Ref: 256). Convenient sampling method was used. Data entry and analysis was done on Statistical Package for Social Sciences version 23. Point estimate at 95% Confidence Interval was calculated along with frequency and proportion for binary data.

**Results:**

Out of 1608 pediatric admissions taken in our study, the prevalence of leave against medical advice was found to be 67 (4.2%) at 95% Confidence Interval (3.22-5.18). Maximum 22 (33%) and minimum 6 (9%) patients respectively belonged to the age group from birth to 7 days and more than 10 years. Out of 67 cases, there were 36 (54%) males and 31 (46%) females.

**Conclusions:**

The prevalence of leave against medical advice among admitted pediatric patients in our study was similar to that of other studies. It is a social health problem which can be prevented by increasing the awareness and facilitating the use of health insurance schemes. More effective communication is required between the treating physicians and the parents to prevent this detrimental practice.

## INTRODUCTION

Clinicians are legally and morally obliged to provide health care services to patients from admission to the end of the treatment process.^1,3^ Patients leaving the hospital during the course of uncompleted treatment or rejecting further treatment are known as "Leave Against Medical Advice" (LAMA). It accounts for 1-26% of discharges.^3-5^

Pediatric patients on LAMA are of concern and challenging to health care providers as children are vulnerable, unable to speak for themselves and it is the wish of the parents/guardians to leave the hospital on their own responsibility despite being counseled about the consequences of LAMA. Researches have shown that LAMA is associated with higher morbidity and mortality.^6^ LAMA is an important issue for doctors and hospital administration and needs to be addressed to prevent it.

This study aimed to find out the prevalence of leave against medical advice among pediatric patients admitted in a tertiary teaching hospital in Nepal.

## METHODS

This is a descriptive cross-sectional study conducted in the pediatric ward of Manipal Teaching Hospital, Pokhara, Nepal from August 2019 to July 2020. Ethical approval for this study was obtained from the institutional review committee (with IRC number 256) of Manipal College of Medical Sciences prior to the initiation of the study.

The sample size was calculated using the following formula:

n = Z^2^ × p × q / e^2^

  = (1.96)^2^ × 0.5 × (1 - 0.5) / (0.03)^2^

  = 1,067

Where,

n = minimum required sample size
Z = 1.96 at 95% Confidence Interval
p = prevalence taken as 50% for maximum sample size
q = 1-p
e = margin of error, 3%

The calculated sample size was calculated to be 1,067. Adding 10% as a non-response rate, the minimum required sample size was 1,174. However, we took the data from 1,608 pediatric patients. Convenient sampling method was used.

All consecutive patients who left against medical advice were included in this desciptive cross-sectional study. We considered the patient to have left against medical advice when the parent/guardian signed and gave their thumb prints on a procedural form acknowledging that they were taking their child on their own accountability and that they were accepting all responsibilities for withdrawing further medical support against the pediatricians' advice. Those who did not give permission to be included from the study were excluded. Data with relevant information about the patient including name, age, sex, number of family members including siblings, health insurance status, diagnosis, number of days admitted, previous admissions if any, timing of LAMA (official working hours from Sunday to Friday from 8:30 am-4:30 pm or during on call hours from Sunday to Friday from 4:30 pm to 8:30 am or on Saturday) were documented. Information pertaining to the parent/guardian signing the LAMA was also taken regarding the relation to the patient, educational status, and employment. Finally the exact reason for the LAMA was sought.

Data was analyzed using the Statistical Package for the Social Sciences (SPSS) version 23 using frequencies, percentages, mean and standard deviation.

## RESULTS

Out of 1608 pediatric admissions, the prevalence of leave against medical advice among pediatric patients in our study was found to be 67 (4.2%) at 95% Confidence Interval (3.22-5.18).

The age of the patients leaving on LAMA ranged from day one of life to 14 years of age. Out of 67 patients, 22 (33%), 7 (10%), 17 (25%), 15 (22%) and 6 (9%) patients belonged to the age group upto 7 days, 8 days-1 month, 1 month-1 year, 1-10 years and 10-16 years. From a total of sixty-seven cases which were identified as LAMA cases, there were 36 (54%) males and 31 (46%) females. The male to female ratio of patients leaving on LAMA was 1.2:1. Twenty two (33%), 24 (36%) and 21 (31%) patients belonged to Brahmin/Chhetri, Janajati and Dalit community. The data regarding the education and employment status of the parents signing LAMA, health insurance of the patients etc. were also taken. Out of all the patients leaving on LAMA, only one had health insurance (Table 1).

**Table 1:** Characteristics of pediatric patients and their guardians in relation to LAMA.

Characteristics	Frequency n (%)
**Age**	
Upto 7 days	22 (33)
8 days- 1 month	7 (10)
1 month - 1 year	17 (25)
1-10 years	15 (22)
10-16 years	6 (9)
**Sex**	
Male	36 (54)
Female	31 (46)
**Ethnicity**	
Brahmin/Chhetri	22 (33)
Janajati	24 (36)
Dalit	21 (31)
Family Members, Median (IQR)	4 (6,3)
**Education of Guardian signing the LAMA**	
Illiterate	7 (10)
Basic	14 (21)
Secondary	39 (58)
University level	7 (11)
**Employment of Guardian**	
Unemployed	4 (6)
Home-maker	15 (22)
Service	13 (19)
Business	7 (11)
Labor work	28 (42)
**Admission in**	
NICU	29 (43)
PICU	16 (24)
Ward	22 (33)
**Health Insurance**	
Yes	1.5 (1)
No	98.5 (66)

**Figure 1:**
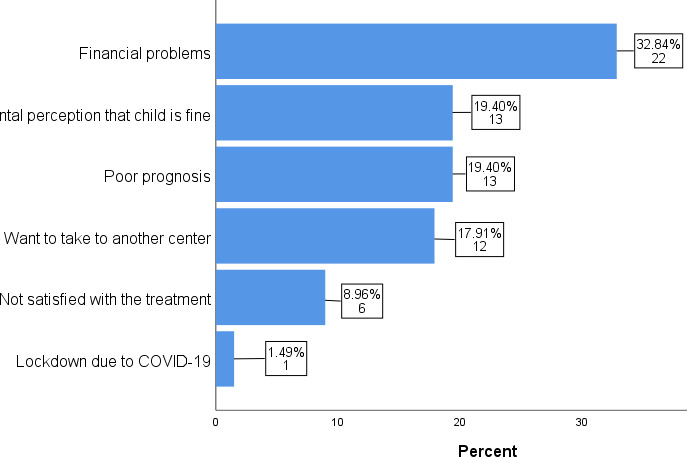
Reasons for Leave against medical advice.

There were various reasons for LAMA such as financial problems in 22 (32.84%), parental perception that the child is fine in 13 (19.40%), poor prognosis in 13 (19.40%), want to take to another center in 12 (17.91%), lack of satisfaction with the treatment in 6 (8.96%) and lockdown due to COVID-19 in 1 (1.49%) (Figure 1).

Financial problems ital perception that child is fine Poor prognosis Want to take to another center Not sati sfied with the treatment Lockdown due to COVID-19

Decision makers and signatories for LAMA were fathers in 40 (59.7%) of cases, mothers in 18 (26.9%) cases, and the rest 9 (13.4%) were other relatives. Among the LAMA cases the duration of hospital stay ranged from 1-11 days with a mean of 3.4±2.6 days (Males: 3.6±2.6 days, Females: 3.1±2.5 days). Most of the patients, 52 (78%) left against medical advice during the official working hours (8:30-16:30 on week days) whereas only 15 (4%) left on weekends (Saturdays). Rest of the patients left during morning and evening hours of week days.

## DISCUSSION

The occurrence of LAMA among pediatric patients poses quite a challenge for pediatricians as they are conflicted between respecting the parents' wishes and complying with their desire to provide maximum care to their vulnerable patients. Most of the LAMA research in children has been studied in countries like Nigeria, Iran and Oman and this research is probably the first in Nepal to include all pediatric patients leaving on LAMA that has been carried out in a pediatric tertiary care center.

The overall rate of LAMA seen in our center was 4.2% of all admissions in pediatric patients which is higher than the rates of 1-2% reported in studies done in adult patients.^7-11^ The higher rate in the pediatric population could be explained by the fact that they are subjected to decisions made by their guardians/parents as they are the ones responsible for the child.

The rate of LAMA in our study is higher than in developed countries like Australia, but lower than in a study done in Kuwait (5.4%) and Iran (8.49%).^7,12,13^ The lower LAMA prevalence possibly reflects the better health services provided by advanced countries.

In the current study, LAMA was observed to be the highest in the neonatal age group and this pattern was noted in studies done from Nigeria.^5,14-16^ This may be accounted by the fact that most of these neonates in our study who left on LAMA had received poor prognostic counseling.

Studies done in Iran and Nigeria showed that LAMA was comparatively higher in males.^5,12^ Surprisingly, there was no significant difference in prevalence of LAMA in relation to gender in our setting considering that the male child is given more privileges in our society in Nepal as it is perceived that males are means of social security for the future. Perhaps our findings indicate that opinions are changing in our society and females too are being given equal opportunities.

Majority of the LAMA occurred in the weekdays during the official working hours between 8:30am to 16:30pm in our study. This was in contrast to the findings of El Malek et al where most of their pediatric patients left on weekends.^13^ Its seems more logical to leave during official duty hours since counseling is done during this time by the senior consultants who are present in the hospital, and decision to leave is most frequently made after the counseling of the condition of the patient which also involves informing the patient's parents regarding the prognosis of the patient.

Financial constraint has been the major reason cited as the reason for LAMA in most studies.^5,14,16,17^ Our study also identified financial problems being the commonest reason for leaving the hospital against medical advice as this was the top most reason cited for LAMA in 32.84% of the cases. Considering that most developing countries have weak and ill managed health insurance schemes, it is no surprise that parents find it difficult to pay for medical treatment out of their own pockets. In our study, only one of the family leaving on LAMA had health insurance whereas in the rest of the cases, funding was done by the parents/guardians. Other major reasons cited in descending order were: parental perception that the child is well and fit to go home, poor prognosis, wanting to take the child to another center for treatment and dissatisfaction with the treatment. These reasons are similar to the ones cited in other similar studies though the order of importance does vary.^12,13,17^

In a study done by El Malek et al, dissatisfaction with the treatment was cited as the most cited reason for LAMA, whereas in our study, it was reported by about 9% of the cases leaving on LAMA.^13^ It is perhaps possible to lower this incidence even further in our setting by promoting better and constant communication between the parents and the treating physicians which may help reduce parent's decision to leave precipitously.

Signatories for LAMA was the father in almost 60% of the cases in the present study and a similar pattern was observed in other countries where the male is usually the decision maker and gender discrimination is recognized.^5,18,19^

One of the limitations of our study was that it was a single institute based study and a multi-centered study comparing reasons for LAMA in teaching hospitals verses non-teaching hospitals is needed. We also did not use a control group in our study comprising of a group who were discharged by the pediatrician after completion of the treatment. Use of this control group would have further strengthened our comprehension and helped us in calculating the predictors of LAMA. Thirdly, follow-up on the LAMA cases were not carried out in this study so, the knowledge of the outcome of the patients leaving on LAMA was not known.

## CONCLUSIONS

We reiterate the need for health insurance schemes covering particularly children and the need for public awareness regarding how these insurance schemes can be availed. Understanding the risk factors for LAMA will also enable pediatricians to develop better preventive plans against LAMA.
